# E-cadherin genetic variants predict survival outcome in breast cancer patients

**DOI:** 10.1186/s12967-016-1077-4

**Published:** 2016-11-16

**Authors:** Hager Memni, Yosra Macherki, Zahra Klayech, Ahlem Ben-Haj-Ayed, Karim Farhat, Yassmine Remadi, Sallouha Gabbouj, Wijden Mahfoudh, Nadia Bouzid, Noureddine Bouaouina, Lotfi Chouchane, Abdelfattah Zakhama, Elham Hassen

**Affiliations:** 1 Laboratory of Molecular Immuno-Oncology, Faculty of Medicine of Monastir, Monastir University, 5019 Monastir, Tunisia; 2 Faculty of Sciences of Bizerte, Carthage University, Bizerte, Tunisia; 3 Higher Institute of Biotechnology of Monastir, Monastir University, Monastir, Tunisia; 4 Cancer Research Chair, College of Medicine, King Saud University, Riyadh, Saudi Arabia; 5 Department of Cancerology and Radiotherapy, Farhat Hached University Hospital, Sousse University, Sousse, Tunisia; 6 Laboratory of Genetic Medicine and Immunology, Weill Cornell Medicine-Qatar, Education City, Qatar Foundation, Doha, Qatar; 7 Department of Anatomy and Pathologic Cytology, Fattouma Bourguiba University Hospital, Monastir University, Monastir, Tunisia

**Keywords:** E-cadherin, SNP, Prognosis, Breast cancer

## Abstract

**Background:**

E-cadherin is a major component of adherens junctions that regulates cell shape and maintains tissue integrity. A complete loss or any decrease in cell surface expression of E-cadherin will interfere with the cell-to-cell junctions’ strength and leads to cell detachment and escape from the primary tumor site. In this prospective study, three functional single nucleotide polymorphisms (−347G/GA, rs5030625; −160C/A, rs16260; +54C/T, rs1801026), were found to modulate E-cadherin expression.

**Methods:**

577 DNA samples from breast cancer (BC) cases were genotyped by polymerase chain reaction-restriction fragment length polymorphism (PCR–RFLP).

**Results:**

We detected no significant correlations between each polymorphism and the clinical parameters of the patients whereas the GACC haplotype was significantly associated with low SBR grading. Overall survival analysis showed that both −347G/G and +54C/C wild (wt) genotypes had a significantly worse effect compared to the other genotypes (non-wt). Moreover, carrying simultaneously both the −347 and +54 wt genotypes confers a significantly higher risk of death. However, with metastatic recurrence, the death-rate was null in patients carrying the non-wt genotypes, and attained 37% in those carrying the wt genotype. A multivariate analysis showed that these two polymorphisms are independent prognostic factors for overall survival in BC patients.

**Conclusions:**

Our results support the fact that E-cadherin genetic variants control disease severity and progression and could be a marker of disease outcome. These findings could be useful in selecting patients that should be monitored differently.

## Background

In 2012, one out of four cancer cases is a breast cancer (BC) case [1]. In North African countries BC is also the commonest cancer among women, and the incidence rates are widely lower than in European Mediterranean countries [2]. The incidence is probably underestimated in these developing countries because of the difficulties related to screening and diagnostic programs. However, considering death rates, BC is the second cause of cancer death in more developed regions (198,000 deaths) and remains the most frequent in less developed regions (324,000 deaths). Yet, whatever socioeconomic status, it is well known that BC is a multifactorial disease, and that besides environment and lifestyle, the genetic background contributes to the increase of the risk of having breast cancer. While a huge amount of studies reported the involvement of genes, genetic loci or genetic polymorphisms in breast cancer susceptibility, studies of the genetic influence on disease progression and severity are less frequent [3]. Defining in gene-candidates, single nucleotide polymorphisms (SNPs) associated with increased severity or worsening progression of BC will potentially allow a better individualized handling of patients and as well as a better understanding of the mechanisms of cancer progression [4–6].

BC recurrence or metastasis represents the main cause of breast cancer-related deaths. It has been shown that epithelial-mesenchymal transition (EMT), a process by which epithelial cells acquire mesenchymal stem cell proprieties, plays a critical role in promoting metastasis in carcinomas [7]. One of the initiating steps of EMT involves downregulation and relocation of the main epithelial cell adhesion protein, the epithelial-cadherin (E-cadherin) [8]. In normal epithelial tissues, E-cadherin is a major component of adherens junctions that regulates cell shape and maintains tissue integrity [9]. A complete loss of E-cadherin expression/function or any decrease in cell surface expression, caused by mutation of the *CDH1* gene, or other mechanisms that decrease E-cadherin expression will interfere with the cell-to-cell junctions’ strength and leads, inter alia, to cell detachment and escape from the primary tumor site. The *CDH1* gene (OMIM 192090) is located on chromosome 16q22, several single nucleotide polymorphisms (SNP) localized in non-coding sequences and affecting protein expression were described. The −*347G/GA* (rs5030625) and −*160C/A* (rs16260) SNPs within the promoter region are the most extensively studied *CDH1*-SNPs in disease association studies. Both the minor alleles were previously shown to reduce E-cadherin expression by affecting the transcriptional efficiency of the CDH1 gene. The −*347GA* allele has weak transcriptional factor-binding strength and transcriptional activity compared with that of the G allele [10], while the −*160A* allele decreases the transcriptional efficiency compared with that of the C allele [11]. Additional regulatory polymorphisms outside of the promoter region that influence E-cadherin expression have also been reported. The +*54C/T* (rs1801026) is located at 141 bp upstream of the poly-A signal in the 3′-UTR region. The study of Jacobs et al. showed that the occurrence of the T allele is related to a lower mRNA stability and also a reduced luciferase expression by a reporter gene constructs driven by a constitutive SV40-promoter [12]. In regards to breast cancer, few investigations have been carried out on the association between *CDH1* polymorphisms and cancer severity or progression. This is why in this prospective cohort study conducted on 577 sporadic BC cases, three functional SNPs from of the E-cadherin gene (*CDH1*) were genotyped, and the possible prognostic values of these genetic variations were investigated.

## Methods

### Study population

The study was approved by the National Ethical Committee and a written informed consent was obtained from all enrolled individuals prior to their participation. Both patients and controls were collected between 2000 and 2012 from the Farhat Hached University Hospital of Sousse (Tunisia). During blood sampling, all participants (patients and controls) were interviewed using a questionnaire to collect demographic characteristics, personal and family medical history, contraceptive methods, sexual and reproductive behavior information and lifestyle (smoking, alcohol consumption). In this study, unrelated participants without family history of breast cancer or any cancer were included. A total of 577 breast cancer patients were recruited from the Department of Cancerology and Radiotherapy (Farhat Hached University Hospital, Sousse, Tunisia). A detailed description of the clinico-pathological characteristics of this cohort is presented in Table 1. The patients had a mean age of 48.7 ± 11 years (range 23–81). The median follow-up was 70 months (range 1–144 months). At the time of this study, 145 patients relapsed after treatment (local or distant recurrence). Among them, 31 patients died from breast carcinoma. To estimate linkage disequilibrium (LD), 300 Tunisian healthy blood donors without a personal history of cancer were recruited as control group from the Regional Center of Blood Transfusion (Farhat Hached University Hospital, Sousse, Tunisia).

**Table 1:** Clinical characteristics of breast cancer patients

Characteristics	Patients (%)
Total	577
Age at diagnosis	
≤40	133 (23.1)
>40	444 (76.9)
Menopausal status	
Non menopausal	298 (51.6)
Menopausal	279 (48.4)
Tumor size	
T1–T2	381 (66)
T3–T4	167 (29)
Unknown	29 (5)
Lymph node involvement	
Negative	230 (39.9)
Positive	328 (56.8)
Unknown	19 (3.3)
Metastasis	
Negative	362 (62.7)
Positive	10 (1.7)
Unknown	205 (35.5)
SBR grade	
SBR 1–2	329 (57)
SBR 3	192 (33.3)
Unknown	56 (9.7)
Histology	
Ductual	513 (88.9)
Lobular	23 (4)
Other	25 (4.3)
Unknown	16 (2.8)
Estrogen receptor	
Negative	195 (33.8)
Positive	255 (44.2)
Unknown	127 (22)
Progesteron receptor	
Negative	240 (41.6)
Positive	210 (36.4)
Unknown	127 (22)
Her-2 status	
Negative	75 (13)
Positive	39 (6.8)
Unknown	463 (80.2)

### Genomic DNA extraction and SNP genotyping analysis

Genomic DNA was extracted from peripheral blood leukocytes by a “salting out” procedure [13]. Briefly, 10 ml of blood was mixed with Triton lysis buffer (0.32 M sucrose, 1% Triton X-100, 5 mM MgCl_2_, 10 mM Tris–HCl, pH 7.5). The pellet was incubated with proteinase K at 56 °C and subsequently salted out using a saturated NaCl solution. Precipitated proteins were removed by centrifugation. The DNA in supernatant fluid was precipitated with ethanol. Finally, the DNA pellet was conserved in Tris–EDTA buffer. DNA concentration and quality were analyzed by thermo-scientific NanoDrop 2000™.

Genotype analysis of the *CDH1* gene SNP −*347 G/GA* (rs5030625), −*160C/A* (rs16260) and +*54 C/T* (rs1801026) polymorphisms were performed by polymerase chain reaction-restriction fragment length polymorphism (PCR–RFLP) using previously described primers (Table 2) [14, 15]. Each PCR was performed under the standard conditions and the amplification was carried out in a final volume of 30 μl containing 25–100 ng of genomic DNA samples, 0.6 μM of each primer for the −160C/A and −347G/GA SNPs and 0.3 μM of each primer for the +54C/T SNP, 0.2 mM desoxy-nucleotide tryphosphate (dNTP), 1.5 mM MgCl_2_, 3 μl of 10 X PCR buffer and 0.5U of SuperTaq DNA polymerase (Amersham, Paris, France). Reaction conditions used with thermal cycler (Biometra, Göttinger, Germany)were as follows: For the promoter SNPs −*160C/A* and −347G/GA the DNA was initially denatured for 5 min at 94 °C for 1 cycle, and incubated for 30 cycles: denaturing for 30 s at 94 °C, annealing for 30 s at 61 °C and extending for 60 s at 72 °C. The +*54C/T* PCR reaction was carried out in the same conditions described previously except for the annealing temperature, that was at 58 °C. A final extension of 7 min at 72 °C was performed at the end of each reaction. After amplification, PCR products were digested at 37 °C over night with 5U of the restriction enzyme *HphI* for the −*160C/A* SNP, for 3 h with 3U of the restriction enzyme *BanII* for the −*347G/GA* and over night with 2U of the restriction enzyme *PmlI* for the +*54C/T* SNP. Digestion products were, then, separated on a 3% agarose gel stained with ethidium bromide and visualized with ultraviolet light.

**Table 2:** Primers and restriction enzymes used for polymorphism genotyping

SNPs	Primer sequences	Restriction enzymes	Fragments size (bp)
−347 G/GA rs5030625 −160 C/A rs16260	Forword: 5′-GCCCCGACTTGTCTCTCTAC-3′ Reverse: 5′-GGCCACAGCCAATCAGCA-3′	*Ban*II	G: 263 + 116 + 68 GA: 332 + 116
		*Hph*I	C: 181 + 177 + 89 A: 266 + 181
+54 C/T rs1801026	Forword: 5′-CAGACAAAGACCAGGACTAT-3′ Reverse: 5′-AAGGGAGCTGAAAAACCACCAGCCAC-3′	*Pml*I	C: 146 + 26 T: 172

### Statistical analysis

To evaluate if our study population (patients and controls) is in the Hardy–Weinberg equilibrium we used the Chi square test to compare between observed and expected genotype frequencies of *CDH1* gene polymorphisms. The same test was used to evaluate any significant association between the three *CDH1* polymorphisms and the clinicopathological characteristics of the disease. The differences were considered significant if the *p* value did not exceed 0.05. Odd ratios (ORs) and 95% confidence intervals (CIs) were calculated by unconditional logistic regression. When expected values in contingency tables were under 5, Fisher’s exact test was used. The LD between SNPs pairs was quantified using the standardized linkage disequilibrium coefficient (D’) [16]. The haplotypes and their frequencies were estimated using the Phase program [17].

Disease-free survival (DFS), metastasis-free survival (MFS), and overall survival (OS) were calculated using the Kaplan–Meier method for ten years and compared with the log-rank test. DFS was defined as the date of diagnosis until first recurrence, metastasis, death due to breast cancer or the last date of follow-up. MFS was defined as the date of diagnosis until first metastasis or last date of follow-up and OS was defined as the date of diagnosis until death due to breast cancer or last date of follow-up. Variables with a p-value less than 0.1 in the univariate Cox regression model were evaluated in a multivariate Cox regression model using the enter method. Because of the low number of the cases with metastasis at diagnosis (n = 10) we choose to exclude the metastasis parameter from the haplotypes and multivariate analysis. All statistics were carried out using Software Package for Social Sciences (SPSS) version 20.0 (SPSS, Chicago, IL, USA).

## Results

### *CDH1* SNPs and their association with clinicopathological characteristics of breast cancer patients

All DNA samples were successfully genotyped for the three *CHD1* SNPs (577 patients and 300 controls). For both patient and control groups all genotype distributions did not diverge significantly from Hardy–Weinberg equilibrium. Moreover, the minor allele frequencies (MAF) of these variants in the control population were close to those reported in Europeans (HapMap consortium) (−347GA: 0.150; −160A: 0.322 and +54T: 0.206). Among the patients, the −*347G/GA* genotype frequencies were 72.4% for *GG*, 24.8% for *GGA* and 2.8% for *GAGA*. For the −*160C/A* SNP, genotype frequencies were 45.9% for *CC*, 43.7% for *CA* and 10.4% for *AA*. The +*54C/T* genotype frequencies were 35.6% for *CC*, 47.1% for *CT* and 17.3% for *TT*.

We then investigated the association between *CHD1* SNP genotype distributions and clinicopathological characteristics at diagnosis of patients with breast cancer. For each SNP genotype analysis, the patients were divided into homozygous wild-type carriers (wt) and non-carriers (non-wt, heterozygous and homozygous mutant). The relationships between *CHD1* SNP genotypes and clinicopathological characteristics are shown in Table 3. Overall, no statistically significant association was observed with any of the three SNPs and clinical characteristics, including age, menopausal status, tumor size, lymph node involvement, metastasis, SBR grading and histologic type.

**Table 3:** Correlation between *CDH1* SNPs and clinicopathological characteristics of breast cancer patients

Characteristics	−*347 G/GA*				−*160 C/A*				+*54 C/T*			
	*GG* ^b^	*GGA* + *GAGA*	*P*	*OR (95% CI)*	*CC* ^b^	*CA* + *AA*	*P*	*OR (95% CI)*	*CC* ^b^	*CT* + *TT*	*P*	*OR (95% CI)*
Age at diagnosis												
≤40	96	37			61	72			48	85		
>40	322	122	0.93	0.98 (0.63–1.51)	204	240	0.98	0.99 (0.67–1.47)	157	287	0.87	1.03 (0.68–1.54)
Menopausal status												
Non menopausal	225	73			145	153			107	191		
Menopausal	193	86	0.08	1.37 (0.95–1.98)	120	159	0.17	1.25 (0.90–1.74)	98	181	0.84	1.03 (0.73–1.45)
Tumor size												
T1–T2	279	102			166	215			134	247		
T3–T4	119	48	0.63	1.1 (0.73–1.65)	81	86	0.28	0.82 (0.56–1.18)	61	106	0.76	0.94 (0.64–1.37)
Lymph node involvement												
Negative	167	63			116	114			73	157		
Positive	239	89	0.94	0.98 (0.67–1.44)	141	187	0.08	1.34 (0.96–1.80)	127	201	0.09	0.73 (0.51–1.05)
Metastasis												
Negative	264	98			166	196			121	241		
Positive	8	2	0.46^a^	0.67 (0.14–3.20)	3	7	0.25^a^	1.97 (0.50–7.70)	2	8	0.37^a^	2 (0.42–9.60)
SBR grade												
SBR 1–2	232	97			150	179			112	217		
SBR 3	148	44	0.10	0.71 (0.47–1.07)	91	101	0.69	0.93 (0.65–1.32)	74	118	0.30	0.82 (0.56–1.19)
Histology												
Ductual	376	137			240	273			183	330		
Lobular	18	5	0.59	0.76 (0.27–2.09)	11	12	0.92	0.95 (0.41–2.21)	6	17	0.35	1.57 (0.60–4.05)

*CI* confidence interval, *OR* odds ratio

^a^Fisher’s exact test

^b^Reference group

To analyze the association of the combined effects of the *CDH1* SNPs and clinicopathological characteristics of patients with breast cancer, *CDH1* haplotype frequencies and linkage disequilibrium coefficient were estimated. The haplotype frequencies among both patients and controls are summarized in Table 4. Eight different haplotypes were observed among both controls and patients with breast cancer. The −*347G*-*160C* + *54C* haplotype was the most frequent among both controls and patients. The −*347G* − *160A* + *54T* haplotype was significantly more frequently observed in patients than controls, and seemed to be a risk haplotype for BC occurrence (p < 10^−4^, OR = 4.03). However, further larger population-based case–control studies are necessary to validate these findings. The LD analysis results showed different patterns between cases and controls. Both −*347G/GA* and −*160C/A* loci, and −*160C/A* and +*54C/T* loci showed a stronger LD in controls compared to patients (*D′* = 0.484 versus *D′* = 0.279 and *D′* = 0.473 versus *D′* = 0.070). Moreover, the −347G/GA and +54C/T loci showed a higher LD in controls compared to patients (*D′ *= 0.083 versus *D′ *= 0.015). The relationships between *CHD1* SNP haplotypes and clinicopathological characteristics are shown in Table 5. The most frequent haplotype was used as a reference in the correlation analysis. The results showed that the −*347GA* − *160C* + *54T* haplotype was significantly more frequent among the younger women (age ≤ 40) (p = 0.01, OR = 0.37). However, the −*347GA* − *160C* + *54C* haplotype was significantly more frequent among patients aged more than 40 years (p = 0.015, OR = 3.74). The −347GA − 160C + 54C haplotype was also significantly more frequent among women with low SBR grading compared to high SBR grading (p = 0.049, OR = 0.44). There was no more significant correlation of *CDH1* haplotypes with clinical characteristics, including menopausal status, tumor size and lymph node involvement and histological type.

**Table 4:** Haplotype frequencies of *CDH1* SNPs observed in breast cancer patients and controls

Haplotypes			Frequencies		*P*
−*347G/GA*	−*160C/A*	+*54C/T*	Patients	Controls	
*G*	*C*	*C*	0.323	0.405	
*G*	*C*	*T*	0.238	0.265	0.270
*G*	*A*	*C*	0.177	0.147	*<10* ^*−4*^
*G*	*A*	*T*	0.109	0.032	*<10* ^*−4*^
*GA*	*C*	*C*	0.068	0.101	0.380
*GA*	*C*	*T*	0.048	0.024	*0.023*
*GA*	*A*	*C*	0.022	0.021	0.660
*GA*	*A*	*T*	0.012	0.003	0.230

**Table 5:** Correlation between *CDH1* haplotype frequencies and clinicopathological characteristics of breast cancer patients

Characteristics	*GCC* ^a^	*GCT*	*GAC*	*GAT*	*GACC*	*GACT*	*GAAC* and *GAAT*
Age at diagnosis							
≤40	0.358	0.213	0.194	0.084	0.027	0.094	0.030
>40	0.312	0.248	0.173	0.117	0.081	0.032	0.036
*P*		*0.15*	*0.83*	*0.15*	*0.015*	*0.01*	*0.21*
OR (CI 95%)	1	1.38 (0.89–2.13)	1.06 (0.64–1.74)	1.54 (0.85–2.79)	3.74 (1.29–10.82)	0.37 (0.17–0.79)	1.37 (0.82–2.28)
Menopausal status							
Non menopausal	0.354	0.236	0.172	0.105	0.056	0.049	0.028
Menopausal	0.291	0.245	0.182	0.114	0.081	0.043	0.044
*P*		*0.21*	*0.26*	*0.26*	*0.098*	*0.92*	*0.25*
OR (CI 95%)	1	1.26 (0.88–1.81)	1.29 (0.83–2.01)	1.29 (0.83–2.01)	1.69 (0.91–3.16)	1.04 (0.51–2.11)	1.73 (0.64–4.39)
Tumor size							
T1–T2	0.318	0.237	0.192	0.105	0.057	0.051	0.038
T3–T4	0.326	0.242	0.149	0.124	0.085	0.038	0.035
*P*		*0.91*	*0.23*	*0.6*	*0.33*	*0.46*	*0.76*
OR (CI 95%)	1	0.98 (0.65–1.46)	0.73 (0.44–1.21)	1.13 (0.71–1.81)	1.41 (0.71–2.79)	0.74 (0.33–1.66)	0.86 (0.32–2.27)
Lymph node involvement							
Negative	0.320	0.284	0.162	0.084	0.053	0.047	0.050
Positive	0.323	0.208	0.195	0.123	0.080	0.047	0.023
*P*		*0.19*	*0.34*	*0.24*	*0.29*	*0.91*	*0.19*
OR (CI 95%)	1	0.78 (0.54–1.13)	1.26 (0.78–2.04)	1.34 (0.82–2.17)	1.43 (0.74–2.77)	0.96 (0.47–1.95)	0.55 (0.22–1.81)
SBR grade							
SBR 1–2	0.314	0.225	0.167	0.131	0.086	0.047	0.030
SBR 3	0.365	0.230	0.192	0.085	0.040	0.055	0.025
*P*		*0.46*	*0.96*	*0.053*	*0.049*	*0.97*	*0.84*
OR (CI 95%)	1	0.86 (0.57–1.29)	0.99 (0.61–1.61)	0.61 (0.37–1.00)	0.44 (0.19–0.99)	1.01 (0.50–2.05)	0.91 (0.34–2.42)
Histology							
Ductual	0.291	0.231	0.139	0.157	0.122	0.026	0.032
Lobular	0.329	0.245	0.179	0.103	0.060	0.050	0.033
*P*		*0.74*	*0.83*	*0.2*	*0.11*	*0.52*	*0.72*
OR (CI 95%)	1.00	1.12 (0.57–2.22)	0.90 (0.35–2.34)	1.59 (0.78–3.23)	2.06 (0.86–4.94)	0.62 (0.14–2.67)	0.91 (0.54–1.52)

^a^Reference group

*CI* confidence interval, *OR* odds ratio

### *CDH1* SNPs and clinicopathological characteristics associated with survival in patients with breast cancer

**Fig. 1:**
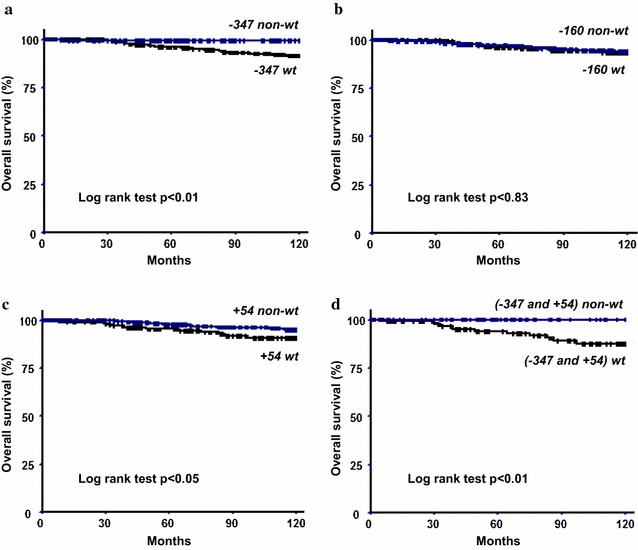
Overall survival curves of breast carcinoma patients according to the presence or absence of the wild type genotypes; **a** −*347G/GA*, **b** −*160C/A*, **c** +*54C/T* and **d** combined −*347G/GA* with +*54C/T*

When the relationship between the genotype distribution of the three SNPs in overall patients and the DFS and MFS was tested, no significant differences were observed (data not shown). However, the 10-years OS curve analysis and log-rank testing showed that both the −*347G/G* and +*54C/C* wild homozygous genotypes had a significantly worse effect on OS compared to the other genotypes (survival rate: 90 versus 95% and 91 versus 99%, respectively) (Fig. 1). Moreover, when patients carrying simultaneously both the −*347G/G* and +*54C/C* genotypes were grouped and compared to the non-carrier patients, the survival rate decreased to 87% for the wt-carriers and increased to 100% for the non-wt carriers (Fig. 1).

**Fig. 2:**
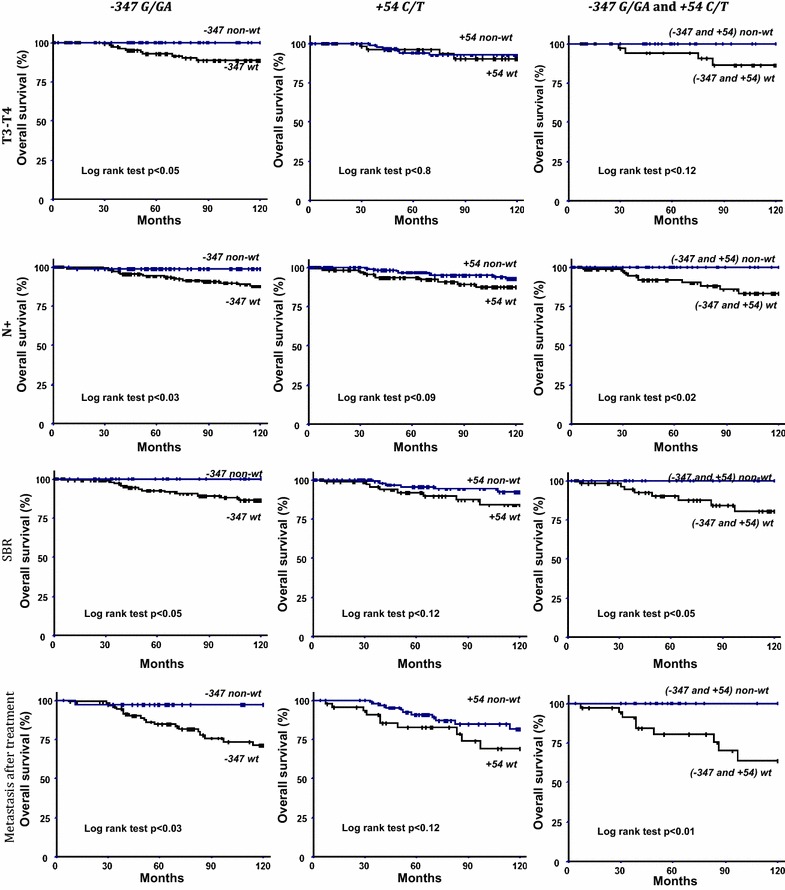
Overall survival curves of different subgroups of breast carcinoma patients according to the presence or absence of the wild type genotypes of −*347G/GA*, +*54C/T* and combined −*347G/GA* with +*54C/T*

Further analyses were conducted to investigate whether the −*347G/GA* and +*54C/T* SNPs were associated with overall survival, according to clinical characteristics of the breast cancer patients (tumor size, lymph node involvement, SBR grading and metastasis occurrence after treatment). Significantly worse survival rates were observed in patients carrying the −*347G/G* wild homozygous genotype among patients with T3–T4 tumor size (survival rates: 88 versus 100%), or positive lymph node involvement (survival rates: 88 versus 99%), or high SBR grading (survival rates: 86 versus 100%) or metastasis occurrence after treatment (survival rates: 71 versus 97%) (Fig. 2). No significant relationship was found with T1–T2 tumor size, negative lymph node involvement, or low SBR grading and non-metastasis occurrence after treatment. Moreover, no significant relationship was found when the +*54C/T* SNP was analyzed according to several patient subgroups. However, when we studied the combined effect of −*347G/G* and +*54C/C* genotypes on overall survival of the positive lymph node involvement (survival rates: 83 versus 100%), high SBR grading (survival rates: 80 versus 100%) and metastasis occurrence after treatment subgroups (survival rates: 63 versus 100%), as noted previously, BC patients carrying simultaneously the wt genotypes had significantly worse survival rates (Fig. 2). Although the results were not statistically significant, when the combined effect was analyzed according to T3–T4 tumor size, they indicated that patients with −*347G/G* and +*54C/C* genotypes had the worst survival rate (survival rates: 86 versus 100%) (Fig. 2).

Univariate and multivariate analyses were used to evaluate whether the *CDH1* SNPs and the clinicopathological characteristics were independent prognostic factors of breast cancer patients. The multivariate analysis showed that tumor size (p = 0.001, HR = 1.80) and lymph node involvement (p = 0.007, HR = 1.66) were independently associated with the DFS (Table 6). Moreover, it showed that lymph node involvement (p = 0.018, HR = 3.26), SBR grade (p = 0.048, HR = 2.20), −*347G/GA* SNP (p = 0.039, HR = 0.12) and +*54C/T* SNP (p = 0.022, HR = 0.40) were independent prognostic factors for OS in breast cancer patients (Table 6).

**Table 6:** Clinicopathological characteristics associated with disease-free survival and overall survival in patients with breast cancer

Characteristics	Disease-free survival						Overall survival					
	Univariate analysis			Multivariate analysis			Univariate analysis			Multivariate analysis		
	HR	95% CI	*P*	HR	95% CI	*P*	HR	95% CI	*P*	HR	95% CI	*P*
Age^a^	0.74	0.51–1.08	0.121	ni			0.75	0.33–1.68	0.487	ni		
Menopausal status^b^	0.92	0.66–1.28	0.638	ni			0.87	0.42–1.76	0.702	ni		
Tumor size^c^	1.87	1.33–2.64	0.0003	1.80	1.27–2.56	0.001	1.44	0.69–3.02	0.326	ni		
Lymph node involvement^d^	1.75	1.22–2.51	0.002	1.66	1.14–2.40	0.007	2.97	1.21–7.30	0.017	3.26	1.22–8.69	0.018
Metastasis^d^	3.03	1.47–6.22	0.003	ni			5.64	1.67–19.01	0.005	ni		
SBR grade^e^	1.09	0.77–1.56	0.599	ni			2.29	1.08–4.84	0.030	2.20	1.00–4.81	0.048
Histology^f^	1.62	0.82–3.20	0.157	ni			0.80	0.11–5.92	0.833	ni		
Estrogen receptor^d^	1.34	0.91–1.95	0.127	ni			0.73	0.33–1.60	0.435	ni		
Progesteron receptor^d^	1.16	0.80–1.67	0.419	ni			0.88	0.40–1.95	0.763	ni		
Her-2 status^d^	1.03	0.44–2.40	0.938	ni			0.67	0.06–7.44	0.746	ni		
*CDH1* −*347 G/GA* ^g^	0.92	0.63–1.35	0.694	ni			0.09	0.01–0.68	0.020	0.12	0.01–0.89	0.039
*CDH1* −*160 C/A* ^g^	0.92	0.66–1.28	0.650	ni			1.27	0.62–2.63	0.505	ni		
*CDH1* +*54 C/T* ^g^	0.98	0.69–1.40	0.951	ni			0.52	0.26–1.06	0.075	0.40	0.18–0.87	0.022

*HR* hazard ratio; *ni* not included in multivariate analysis

^a^≤40 versus >40 years

^b^Non menopausal versus menopausal

^c^T1–T2 versus T3–T4

^d^Negative versus positive

^e^SBR 1–2 versus SBR 3

^f^Ductal invasive carcinoma versus Lobular invasive carcinoma

^g^Homozygous wild allele type versus heterozygous and mutates homozygous allele types

## Discussion

Previous studies have shown that the development and the progression of epithelial cancers such as BC are related to the loss or the reduced expression of the main intercellular adhesion molecule of epithelial cells, the E-cadherin. The loss of cell-to-cell adhesion is an early event in metastatic colonization, leading to the detachment of the cell from her tissue of origin to colonize other sites.

E-cadherin expression is under the control of functional SNPs. As far as we know, reports were mainly case control-studies looking for risk of developing BC, and very few studies with controversial results were investigated to identify the relationship between *CDH1* genetic variants and clinicopathological features of the patients. In our study, none of the studied SNPs showed any significant correlations with patients’ epidemiological or tumor or histological features. Shabnaz et al. also did not find any correlation between −*160C/A* polymorphisms with clinicopathological characteristics of BC patients [18]. However, Tipirisetti et al. noted a positive correlation between the −*160A* allele occurrences in patients with advanced stage [19]. In a Taiwanese study conducted on hepatocellular carcinoma patients, the occurrence of the −*160A* allele was significantly associated with more severe clinical stages [20]. Conversely, in a recent study of pancreatic cancer cases, the −*160AA* genotype was found to be significantly associated with reduced risk with T stage, lymph node metastasis and pathological stage [21]. In accordance with the previous results, a Japanese study of gastric cancer cases found that the −*160CC* genotype was significantly associated with deep invasion and lymph node metastasis [22]. Although previous studies were conducted mainly on epithelial cancer cases, a possible explanation of these contrasting results is the occurrence of one or more other SNPs, found in strong LD with the −*160C/A* in some ethnic groups (e.g. rs7200690, rs9929218). Further studies should be conducted among different ethnic groups to help understanding these results.

To see the combined effects of the three functional SNPs the haplotype analysis was investigated. Weak LD values, generally associated with higher recombination rates, were observed among patients [23]. Conversely, stronger LD values were observed among controls, suggesting a possible protective effect of the strong LD against BC. However, further investigations with a larger control sample size are needed to explain this result. To the best of our knowledge, this is the only study that explored the relationship between *CDH1* haplotype frequencies and tumor severity. The −*347GA* − *160C* + *54C* haplotype may have a protective effect against high SBR grading. This haplotype has only one mutated allele at the −347 position, whilst having protective effect, however, the *GA* allelic analysis did not show significant differences with the *G* allele when patients were compared according to SBR grading (p = 0.12, OR = 0.75). Although each SNP had a functional impact on E-cadherin expression, the effects of their interactions and combinations are unknown, hence there is a need for further studies to describe the functionality of haplotypes including these three SNPs.

Moreover, very few studies investigated the associations of the *CDH1* SNPs with patient survival. In a recent study conducted on a Chinese population, Jia et al. showed that BC patients with low clinical tumor stages and carrying the minor allele genotype of an SNP (rs7200690) located in intron 2 in strong LD with the −*160C/A* SNP, had unfavorable disease-free survival [24]. However, in a British population-based study no effect of the −*160C/A* SNP was seen on BC survival [25]. In the present study the −*347G/GA* and +*54C/T* SNPs, but not the −*160C/A* SNP, were shown to be associated with BC overall survival. When considering the whole patient group, both wild genotypes were shown to be associated with worse BC overall survival. Moreover, patients carrying simultaneously the wild genotypes, −*347G/G* and +*54C/C*, had worse survival rates than those carrying one of each genotype. Interestingly, when considering more aggressive tumor subgroups (T3–T4, lymph node positive, high grade SBR and metastasis occurrence after treatment) we observed significantly worse survival rates with the −*347G/G* genotype and a decrease in survival when patients carry simultaneously the −*347G/G* and +*54C/C* genotypes. Herein we found that the *CDH1* −*347G/G* genotype confer risk of death in patients with more aggressive BC progression.

It is well known that in normal epithelial tissues, E-cadherin expression has suppressive effects on tumor progression, invasion and metastasis and thus any deregulation of E-cadherin expression could have critical pathological consequences. In a pathological context, E-cadherin expression could be modulated by several mechanisms, loss of heterozygosity, mutations of the *CDH1* gene, epigenetic modulation, proteolytic processing and also cadherin switching [9]. In BC, studies on E-cadherin tissue expression were conflicting. Most of them showed that reduced or loss of E-cadherin expression correlates with high histological grade, larger tumor size, nodal metastasis, development of distant metastasis, and a reduced disease-free and overall survival [26–28]. However, in recent reports the involvement of E-cadherin in breast cancer severity and progression has increasingly been suggested [29, 30]. The ambiguous role of E-cadherin could be partially due to the existence of at least two different and functional forms of E-cadherin, a full-length membrane form and an extracellular proteolytic soluble form (sE-cad). During the oncogenic process the first consequence of the E-cadherin proteolysis is the cell detachment and the release of a functional sE-cad with cancer promoting functions. Inside the tumor microenvironment, sE-cad inhibits cell-to-cell adhesion through an efficient competitive manner and by stimulating the activity of multiple matrix metalloproteinases (MMPs) [31]. Then, when sE-cad diffuse and spread into blood circulation, multiple oncogenic signaling pathways are activated [32, 33]. Recently, Liang et al., assessed the clinical significance of serum sE-cad levels in BC patients. They observed a significant correlation of sE-cad levels with tumor stage, grade, lymph node metastasis and also survival [34]. Moreover, Hofmann et al., found that serum sE-cad levels might be a marker predicting response to preoperative chemotherapy for patients with locally advanced breast cancer [35]. Functional SNPs modulate the expression of both the membrane and the soluble E-cadherin forms in the same way. Since the two forms play opposite functions, the membrane form expressed in normal tissue acts as a tumor suppressor, while the soluble form associated with disease severity promotes tumor progression, this could explain why patients expected to have reduced E-cadherin expression have better survival whatever the severity of the disease.

## Conclusions

This study provides novel information about the relationship between E-cadherin (*CDH1*) genetic variants and clinicopathological features and progression of BC. The main finding of this study is the association of *CDH1* functional SNPs with overall survival in BC, particularly in patients with a more aggressive tumor at onset or with recurrent metastatic BC. Our results support the fact that the *CDH1* SNPs control disease severity and progression and could be a marker of disease outcome. These findings could be useful in selecting patients who should be monitored differently. Additional investigations on functional evaluation of *CDH1* SNPs should be carried out to support our findings.

## Abbreviations

BC: breast cancer
PCR-RFLP: polymerase chain reaction-restriction fragment length polymorphism
wt: wild genotype
SNP: single nucleotide polymorphism
EMT: epithelial-mesenchymal transition
E-cadherin: epithelial-cadherin
*CDH1*: *cadherin1* gene
LD: linkage disequilibrium
ORs: odd ratios
CIs: 95% confidence intervals
D’: disequilibrium
DFS: disease-free survival
MFS: metastasis-free survival
OS: overall survival
sE-cad: soluble E-cadherin
MMPs: multiple matrix metalloproteinases
